# Urinary incontinence associated with anxiety and depression: the impact of psychotropic drugs in a cross-sectional study from the Norwegian HUNT study

**DOI:** 10.1186/s12888-020-02922-4

**Published:** 2020-11-02

**Authors:** Gunhild Felde, Anders Engeland, Steinar Hunskaar

**Affiliations:** 1 Department of Global Public Health and Primary Care, University of Bergen, Postboks 7804, 5020 Bergen, Norway; 2 Department of Chronic Diseases and Ageing, Division of Mental and Physical Health, Norwegian Institute of Public Health, Bergen, Norway

**Keywords:** Urinary incontinence, Depression, Anxiety, Psychotropic drugs, Epidemiology

## Abstract

**Background:**

Anxiety and depression are in both cross-sectional and longitudinal studies associated with urinary incontinence (UI) in women, strongest for the urgency component of UI. The role of psychotropic drugs in this association, especially antidepressants, has been questioned, but not clarified. The present study aimed to explore the associations between UI and anxiety/depression and the possible impact of psychotropic drugs on these associations.

**Methods:**

We conducted a cross-sectional, population-based study with questionnaire data from 21,803 women ≥20 years in the Norwegian Nord-Trøndelag Health Study merged with the Norwegian Prescription Database, which contains information on all dispensed prescriptions. We used multivariate logistic regression to investigate the association between UI (any UI, and by type and severity) and anxiety/depression (by different score on Hospital anxiety and depression scale), and the influence of psychotropic drugs on this association (by different volume of drug use).

**Results:**

Compared with normal anxiety- and depression score, having moderate/severe anxiety or depression (HADS≥11) increased the prevalence of UI from 27.6 to 37.8% (OR 1.59 (1.40–1.81), *p* < 0.001) for anxiety and from 28.0 to 43.7% (OR 1.79 (1.46–2.21), p < 0.001) for depression. According to type of UI, mixed UI was most strongly associated with a high HADS-score with an odds ratio 1.84 (1.65–2.05) for anxiety and 1.85 (1.61–2.13) for depression. Compared to no UI, severe UI was associated with depression with odds ratios of 2.04 (1.74–2.40), compared with no UI. Psychotropic drug use did not influence the associations between UI and anxiety/depression. We found high prevalence of UI among users of various psychotropic drugs. After adjustments, only antidepressants were associated with UI, with OR 1.36 (1.08–1.71) for high defined daily dose of the drug. Anxiolytics were associated with less UI with OR 0.64 (0.45–0.91) after adjustments for anxiety.

**Conclusion:**

This study showed that anxiety, depression and use of antidepressants are associated factors with UI, strongest for urgency and mixed type of UI, with increasing ORs by increasing severity of the conditions and increased daily dose of the medication. Use of antidepressants did not influence the associations between UI and anxiety/depression.

## Background

Depression and anxiety are in cross-sectional studies associated with higher prevalence of urinary incontinence (UI) in women [1–4]. According to type, studies show an association to all three main types of UI, but strongest for urgency and mixed UI [3, 5]. In three longitudinal studies, depression at baseline predicted onset of UI [6–8].

The serotonergic and noradrenergic systems are involved in both bladder control and in the pathology of depression/anxiety. Antidepressants affecting this neuro-hormonal system are the leading pharmacological intervention for mood- and anxiety disorders [9–11], but the antidepressant duloxetine has also documented effect as treatment for stress UI [12, 13]. Beside being a treatment for UI, several studies have also shown an association between the use of antidepressants and high prevalence of UI, strongest for mixed and urgency UI [14–16]. It is unknown whether confounding by indication can explain this association, or whether antidepressants induce UI through biological mechanisms.

In the present cross-sectional study, the aim was firstly to investigate the associations between UI and anxiety/depression and psychotropic drug use for different severity of the conditions and for different volume of drug use. Secondly, we aimed to investigate if the associations between UI and anxiety/depression for the possible impact of psychotropic medication use.

## Methods

In this cross-sectional study we used data from the third wave of The Nord-Trøndelag Health Study (HUNT3) and the Norwegian Prescription Database (NorPD).

HUNT3 (2006–08) was a population-based survey inviting all persons ≥20 years in the former county of Nord-Trøndelag (*n* = 47,293 women) [17], which is now part of the county Trøndelag. The survey included questions covering a broad specter of medical conditions. Together with an invitation by post, the participants received a questionnaire (Q1), which they brought to a screening station where they underwent clinical examinations and blood tests. The women received another questionnaire (Q2) at the screening station, which they filled in at home and returned by mail. Q2 included questions about anxiety, depression, and UI. A total of 27,692 (59%) women answered Q1 and received Q2 (source population). 23,141 women answered Q2 and 21,803 (79%) of these answered the UI-part of the questionnaire (study population).

### Assessment of urinary incontinence

The EPINCONT study (Epidemiology of Incontinence in Nord-Trøndelag) is the UI part of the HUNT surveys [18]. We define UI as any leakage of urine [19]. If the participant answered yes on the entry question if she had experienced urine leakage, she was asked more specific questions about frequency (four levels), amount (three levels) and in which situations she experienced the leakage. Those who, despite answering “no” or failing to answer the entry question, answered confirmatively on the specific questions, were also regarded as answering “yes” on the entry question. Those not answering the entry question about leaking urine and answered two or less of the following three questions, where classified as missing (*n* = 40). A stress component was defined if the participant leaked urine when coughing, laughing, sneezing, or making an effort. If she had leakage in conjunction with urgency to void, we defined an urgency component. If answering “yes” on both these questions, we defined the leakage as mixed UI. Those who answered “no” on both the urgency and stress UI question, despite answering “yes” on the entry question about loss of urine, were grouped as unclassified. We used the four-level Sandvik severity index to categorize the severity of the UI [20, 21]: the reported frequency was multiplied by the amount of leakage, resulting in an index value (1–12), further categorized into three or four severity levels. In our analysis three levels were used.

### Assessment of depression and anxiety

High score on the Hospital Anxiety and Depression Scale, HADS-A and HADS-D, defined anxiety and depression, respectively [22–25]. HADS is a self-administered questionnaire with seven questions for anxiety (HADS-A) and seven for depression (HADS-D). Each item has four possible answers, scored on a Likert scale from zero to three, giving subscales from 0 to 21 when added. Zero is minimum and 21 is maximum symptom level on separate scales for anxiety and depression. A substitution of missing data was performed for persons who responded to five or six of the HADS-A and HADS-D questions by assuming similar responses on the questions not answered as in those answered (*n* = 966). When from one to four questions were answered, the score was regarded as missing (*n* = 319). HADS-A and HADS-D score of eight or more defined clinically significant anxiety and depression, respectively. We defined mild anxiety and depression by score 8- < 11 and moderate/severe anxiety and depression by score ≥ 11 on the HADS-A and HADS-D-scale, respectively.

### Assessment of psychotropic medication use

The NorPD is a national health register containing information about all prescriptions dispensed at Norwegian pharmacies. Each time a drug is dispensed, the generic name, Anatomical Therapeutic Chemical (ATC)- code, strength, number of packages and defined daily dose (DDD) are registered. DDD is the average maintenance dose per day for a drug used for its main indication in adults. The DDD reflects the total amount of drug prescribed and dispensed, and the overall amount of the actual drug during the last 6 months was calculated. We defined drug use as dispensed prescription during the last 6 months. In the dose-response analysis, we used four different degrees of DDD: no use, low DDD, medium DDD and high DDD. The cut-offs were set separately for each drug, due to different user profiles. The following drug groups were used in the analyses: Opioid analgesics (ATC-code: N02A), other analgesics (N02B), antiepileptic drugs (N03), lamotrigine (N03A X09), antiparkinson drugs (N04), antipsychotics (N05A), anxiolytics (N05B), hypnotics and sedatives (N05c), antidepressants (N06A) and selective serotonine reuptake inhibitors (SSRIs) (N06AB), urologics (G04BD), estrogens (G03C) and progesterons and estrogens in combination (G03F). We selected drug groups due to literature indicating possible association with UI [13–16, 26–28].

Variables selected from HUNT3 and NorPD were linked, using the identification number assigned to everyone living in Norway.

### Statistical analyses

Level of statistical significance was set at 5%. SPSS version 25 was used for analyses. We used descriptive statistics to characterize the study group regarding UI, anxiety, depression, and medication use.

We used multivariate logistic regression with anxiety and depression as dependent variables to investigate the association between anxiety and UI and depression and UI, respectively, including by different types and severity of UI. To investigate the possible effect modification of psychotropic drug use on anxiety and depression, we used two logistic regression models with UI as dependent variable in both. In the first model, use of different psychotropic drugs by DDD groups were independent variables together with depression. In the other model, use of psychotropic drugs by DDD groups were independent variables together with anxiety. Secondly, we analyzed the prevalence and ORs of UI in groups of individuals with anxiety and depression, comparing users and non-users of antidepressants and anxiolytics. In all the logistic regression analyses, we adjusted for the following potential confounders and risk factors: age, BMI, parity, diabetes, asthma, myocardial infarction, cerebral stroke, chronic pain, rheumatoid arthritis, use of urologic medication for urgency, and use of estrogen replacement medication. The adjustment variables were chosen based on pre-specified known risk factors and medication with possible impact on UI prevalence [16, 29].

## Results

The mean age of women was 53 years, 61% were overweight (BMI 25.0–29.9) or obese (BMI ≥ 30), mean number of children was 2.1. Characteristics regarding UI, anxiety, depression, and medication use are shown in Table 1. 29% of the women met the definition of UI, 23% in the youngest group (19–39 years), 32% in the oldest group (≥55 years). Stress UI was most common type in the two youngest age groups (up to 54 years), mixed was most common in the oldest group. 12% of all women with UI had severe or very severe UI, 19% in the oldest group.

**Table 1:** Characteristics of the study population (*N* = 21,803), by age groups

Age at inclusion (years) Number of women (N)	19–39 ***N*** = 4916		40–54 ***N*** = 6726		≥55 ***N*** = 10,161		All ***N*** = 21,803	
	N	%	N	%	N	%	N	%
**Parity** ^**a**^								
None	1577	32.1	402	6.0	643	6.3	2622	12.0
1	712	14.5	645	9.6	733	7.2	2090	9.6
2	1565	31.8	2770	41.2	3296	32.4	7631	35.0
≥ 3	1050	21.4	2895	43.0	5472	53.9	9417	43.2
**Body mass index** ^**a**^								
< 18.5 (underweight)	72	1.5	40	0.6	70	0.7	182	0.8
18.5–24.9 (normal)	2515	51.2	2707	40.2	2991	29.4	8213	37.8
25.0–29.9 (overweight)	1434	29.2	2579	38.3	4245	41.8	8258	38.0
≥ 30 (obesity)	879	17.9	1387	20.6	2806	27.6	5072	23.3
**Asthma**	668	13.6	752	11.2	1211	11.9	2631	12.1
**Myocardial infarction**	1	0	24	0.4	312	3.1	337	1.5
**Cerebral stroke**	11	0.2	72	1.1	424	4.2	507	2.3
**Diabetes**	47	1.0	137	2.0	672	6.6	856	3.9
**Continence status** ^**a**^								
Continent	3800	77.3	4735	70.4	6936	68.3	15,471	71.0
Any UI	1116	22.7	1991	29.6	3225	31.7	6332	29.0
**Type of UI (*n*** **= 6332)** ^**a**^								
Stress	613	54.9	1015	50.9	1068	33.2	2696	42.6
Urgency	136	12.2	213	10.7	524	16.2	873	13.8
Mixed	296	26.5	705	35.4	1471	45.6	2472	39.0
Other/unclassified	68	6.1	51	2.6	132	4.1	251	4.0
Missing	3	0.3	7	0.4	30	0.9	40	0.6
**Severity of UI (*n*** **= 6332)** ^**a**^								
Slight	664	59.5	959	48.2	952	29.5	2575	40.7
Moderate	398	35.7	888	44.6	1498	46.4	2784	44.0
Severe	33	3.0	89	4.5	522	16.2	644	10.2
Very severe	1	0.1	14	0.7	101	3.1	116	1,8
Missing	20	1.8	41	2.1	152	4.7	213	3.4
**Anxiety**								
**HADS-A 8–10.9**	569	11.6	714	10.6	1106	10.9	2389	11.0
Antidepressant status (*n* = 2389) ^a^								
Using	62	10.9	114	16.0	211	19.1	387	16.2
Not using	507	89.1	600	84.0	895	80.9	2002	83.8
**HADS-A ≥ 11**	311	6.3	442	6.6	563	5.5	1316	6.0
Antidepressant status (*n* = 1316) ^a^								
Using	62	19.9	130	29.4	197	35.0	389	29.6
Not using	249	80.1	312	70.6	366	65.0	927	70.4
**Depression**								
**HADS-D 8–10.9**	215	4.4	377	5.6	839	8.3	1431	6.6
Antidepressant status (*n* = 1431) ^a^								
Using	32	14.9	94	24.9	178	21.2	304	21.2
Not using	183	85.1	283	75.1	661	78.8	1127	78.8
**HADS-D ≥ 11**	60	1.2	146	2.2	240	2.4	446	2.0
Antidepressant status (*n* = 446)) ^a^								
Using	21	35.0	49	33.6	85	35.4	155	34.8
Not using	39	65.0	97	66.4	155	64.6	291	65.2
**Psychotropic drug use** ^**b**^								
Opioid analgesics	245	5.0	540	8.0	912	9.0	1697	7.8
Non-opioid analgesics	136	2.8	385	5.7	891	8.8	1412	6.5
Anti-epileptics	47	1.0	133	2.0	213	2.1	393	1.8
Lamotrigin	21	0.4	26	0.4	35	0.3	82	0.4
Anti-parkinson drug	1	0	7	0.1	87	0.9	95	0.4
Anti-psychotics	34	0.7	107	1.6	238	2.3	379	1.7
Anxiolytics	72	1.5	283	4.2	817	8.0	1172	5.4
Hypnotics/sedatives	91	1.9	391	5.8	1693	16.7	2175	10.0
Anti-depressants (all)	234	4.8	588	8.7	1125	11.1	1947	8.9
SSRI	163	3.3	358	5.3	653	6.4	1174	5.4

*UI* Urinary incontinence

*HADS* Hospital anxiety and depression scale

*SSRI* selective serotonin receptor inhibitor

^a^Column-distribution

^b^ Drug use was defined as dispensed prescription last 6 months

6% met the definition of mild depression, while 2% had moderate/severe depression. In those two groups, 21 and 35% used an antidepressant drug, respectively.

11% had mild anxiety and 6% moderate/severe anxiety. In these groups the use of antidepressant drugs was 16 and 30%, respectively. 9% of all women used antidepressants, 11% in the oldest age group. 60% of the antidepressant users used a SSRI. 10% used hypnotics and sedatives, 17% among the oldest. 5% used an anxiolytic.

Anxiety and depression were both associated with UI, with ORs 1.48 (1.36–1.60) and 1.58 (1.42–1.76), respectively, when adjusted for known confounders and risk factors for UI. Both anxiety and depression were strongest associated with mixed and severe UI. Depression was associated with severe UI, with OR 2.04 (1.74–2.40) (Table 2).

**Table 2:** Odd ratios (ORs) for anxiety (HADS-A ≥ 8) and depression (HADS-D ≥ 8) for women with any urinary incontinence (UI), different types and severities of UI versus continence. *N* = 21,803

	Anxiety		Depression	
	Unadjusted OR	Adjusted* OR	Unadjusted OR	Adjusted* OR
**Continence**	1.00 (Ref.)	1.00 (Ref.)	1.00 (Ref.)	1.00 (Ref.)
**Any UI**	1.46 (1.35–1.57)	1.48 (1.36–1.60)	1.74 (1.58–1.92)	1.58 (1.42–1.76)
**Severity of UI**				
Slight	1.29 (1.16–1.44)	1.31 (1.17–1.47)	1.14 (0.97–1.32)	1.17 (1.00–1.38)
Moderate	1.48 (1.31–1.66)	1.52 (1.34–1.72)	1.78 (1.53–2.07)	1.67 (1.42–1.96)
Severe	1.69 (1.50–1.91)	1.74 (1.52–1.99)	2.61 (2.26–3.00)	2.04 (1.74–2.40)
**Type of UI**				
Stress	1.24 (1.11–1.37)	1.27 (1.13–1.42)	1.37 (1.19–1.58)	1.38 (1.18–1.60)
Urgency	1.32 (1.11–1.58)	1.44 (1.20–1.73)	1.78 (1.44–2.20)	1.50 (1.19–1.90)
Mixed	1.84 (1.66–2.03)	1.84 (1.65–2.05)	2.17 (1.91–2.46)	1.85 (1.61–2.13)

* Adjusted for age, BMI, parity, diabetes, asthma, myocardial infarction, cerebral stroke, chronic pain, rheumatoid arthritis, use of urologic medication and use of estrogen replacement medication

The following psychotropic drug groups were associated with UI in the unadjusted analyses, also with a dose-dependent trend (not shown in tables): antidepressants generally, SSRIs, opioid and other analgesics and antiparkinson drugs. Antipsychotics and hypnotics were associated, without any dose-dependent trend. Anxiolytic or antiepileptic drugs in general were not associated with UI, but the antiepileptic drug lamotrigine was associated with UI, although not in a dose-dependent manner. Table 3 shows the results of two logistic regression models with UI as dependent factor, and anxiety and depression as independent factors, respectively. The results demonstrate the influence of psychotropic drugs on the association between anxiety and UI and depression and UI. After adjusting for all psychotropic drugs, the OR for UI for persons with anxiety did not change compared to only adjusting for the other confounders and risk factors. However, medium and high volume of antidepressants and high volume of antiparkinson drugs were associated with UI. Use of high volume of anxiolytics and medium volume of hypnotics/sedatives were negatively associated with UI. After adjustments for all psychotropic drugs, depression was associated with UI with OR 1.55 (1.39–1.73), compared with OR 1.58 (1.42–1.76) when only adjusting for the other confounders and risk factors. Also, in these analyses medium and high volume of antidepressants were associated with UI. High volumes of anxiolytics and medium volumes of hypnotics/sedatives were negatively associated.

**Table 3:** Odds ratios (ORs) for urinary incontinence (UI) (dependent variable) for persons with high anxiety score- and for persons with high depression-score (HADS-A and HADS-D ≥ 8, respectively) versus normal anxiety-score and normal depression-score (HADS-A and HADS-D < 8, respectively) (independent variables). ORs for the different drug groups by different volume (DDD) of drug use. *N* = 21,803

		Anxiety (HADS-A ≥ 8)		Depression (HADS-D ≥ 8)	
		OR *	OR**	OR*	OR**
Reference (HADS-A/−D < 8)		1.00	1.00	1.00	1.00
Any UI		**1.48 (1.36–1.60)**	**1.48 (1.36–1.61)**	**1.58 (1.42–1.76)**	**1.55 (1.39–1.73)**
Antidepressants (*n* = 1947)	Low DDD (*n* = 866)		1.10 (0.94–1.30)		1.12 (0.95–1.32)
	Medium DDD (*n* = 684)		**1.30 (1.08–1.55)**		**1.31 (1.09–1.56)**
	High DDD (*n* = 397)		**1.36 (1.08–1.71)**		**1.40 (1.12–1.77)**
Opioid analgesics (*n* = 1697)	Low DDD (*n* = 674)		**0.80 (0.67–0.97)**		**0.80 (0.66–0.97)**
	Medium DDD (*n* = 678)		1.00 (0.83–1.20)		1.00 (0.83–1.20)
	High DDD (*n* = 345)		1.08 (0.83–1.40)		1.08 (0.83–1.41)
Other analgesics (*n* = 1412)	Low DDD (*n* = 417)		0.88 (0.69–1.11)		0.88 (0.69–1.11)
	Medium DDD (*n* = 744)		0.98 (0.82–1.17)		0.99 (0.83–1.18)
	High DDD (*n* = 251)		1.18 (0.88–1.58)		1.15 (0.86–1.54)
Antiepileptic drugs (*n* = 393)	Low DDD (*n* = 156)		0.86 (0.58–1.26)		0.85 (0.58–1.26)
	Medium DDD (*n* = 165)		1.21 (0.85–1.73)		1.23 (0.86–1.75)
	High DDD (*n* = 72)		1.13 (0.67–1.92)		1.13 (0.67–1.92)
Antiparkinson drugs (*n* = 95)	Low DDD (*n* = 36)		1.18 (0.55–2.46)		1.18 (0.56–2.50)
	Medium DDD (*n* = 40)		1.35 (0.68–2.92)		1.28 (0.62–2.70)
	High DDD (*n* = 19)		**2.64 (1.02–6.85)**		2.47 (0.94–6.45)
Antipsychotic drugs (*n* = 379)	Low DDD (*n* = 153)		1.38 (0.96–1.98)		1.40 (0.98–2.01)
	Medium DDD (*n* = 143)		0.97 (0.66–1.44)		0.97 (0.66–1.44)
	High DDD (*n* = 83)		1.14 (0.69–1.89)		1.15 (0.70–1.90)
Anxiolytic drugs (*n* = 1172)	Low DDD (*n* = 449)		0.89 (0.71–1.11)		0.94 (0.75–1.17)
	Medium DDD (*n* = 485)		0.80 (0.64–1.00)		0.86 (0.69–1.07)
	High DDD (*n* = 238)		**0.64 (0.45–0.91)**		**0.67 (0.48–0.95)**
Hypnotic and sedative drugs (*n* = 2175)	Low DDD (*n* = 930)		0.96 (0.82–1.13)		0.99 (0.84–1.15)
	Medium DDD (*n* = 827)		**0.79 (0.66–0.94)**		**0.80 (0.67–0.95)**
	High DDD (*n* = 418)		1.00 (0.79–1.28)		1.02 (0.80–1.30)

*Adjusted for age, BMI, parity, diabetes, asthma, myocardial infarction and cerebral stroke, chronic pain, rheumatoid arthritis, use of urologic medication and use of estrogen replacement medication. ** Adjusted for the factors* and **i**n addition psychotropic drugs. DDD: defined daily dose

Table 4 shows the prevalence of UI and odds ratios for UI for women with increasing HADS-A- and HADS-D-score and for women with high HADS-scores using antidepressants or anxiolytics compared with non-users. For persons with no anxiety, mild, or moderate/severe anxiety, the prevalence of UI was 28, 35 and 39%, respectively. For persons with anxiety using antidepressants, the prevalence of UI was 41%, not significant after adjustments, when compared to the women with anxiety not using antidepressants, OR 1.11 (0.93–1.34). For persons with anxiety, using anxiolytics was associated with a small, but significant decrease in UI, OR 0,80 (0,64-0,99). The same decrease in OR was present among depressed women using an anxiolytic, but with lack of statistical significance. For persons with no depression, mild, or moderate/severe depression the prevalence of UI increased from 28, to 39% and 44%, respectively, but there was no additional increase in prevalence in the depressed group using antidepressants.

**Table 4:** Prevalence of and odds ratio for urinary incontinence (UI) for women with and without anxiety and depression, and women with anxiety and depression using anxiolytics or antidepressants compared to the women with anxiety and depression not using anxiolytics or antidepressants. *P*-values by chi-quadrat test

	n	%	P	Unadjusted OR	Adjusted^**a**^ OR
**Anxiety (*n*** **= 21,511)**			< 0.001		
HADS-A < 8	4922	27.6		1.00 (Ref.)	1.00 (Ref.)
HADS-A 8–10.9	827	34.6		**1.39 (1.27–1.52)**	**1.42 (1.29–1.56)**
HADS-A ≥ 11	498	37.8		**1.59 (1.42–1.79)**	**1.59 (1.40–1.81)**
**HADS-A ≥ 8 (*n*** **= 3705)**					
Without use of antidepressant	1008	34.4	0.001	1.00 (Ref.)	1.00 (Ref.)
With use of antidepressant	317	40.9		**1.32 (1.12–1.55)**	1.11 (0.93–1.34)
Without use of anxiolytic	1138	35.9	0.72	1.00 (Ref.)	1.00 (Ref.)
With use of anxiolytic	187	35.1		0.97 (0.80–1.17)	**0.80 (0.64–0.99)**
**Depression (*n*** **= 21,545)**	21,545		< 0.001		
HADS-D < 8	5504	28.0		1.00 (Ref.)	1.00 (Ref.)
HADS-D 8–10.9	562	39.3		**1.66 (1.49–1.86)**	**1.52 (1.35–1.72)**
HADS-D ≥ 11	195	43.7		**2.00 (1.65–2.42)**	**1.79 (1.46–2.21)**
**HAD-D ≥ 8 (*n*** **= 1877)**					
Without use of antidepressant	556	39.2	0.08	1.00 (Ref.)	1.00 (Ref.)
With use of antidepressant	201	43.8		1.21 (0.98–1.50)	1.08 (0.85–1.37)
Without use of anxiolytic	648	40.5	0.76	1.00 (Ref.)	1.00 (Ref.)
With use of anxiolytic	109	39.5		0.96 (0.74–1.25)	0.85 (0.63–1.14)

^a^Adjusted for age, BMI and parity, diabetes, asthma, myocardial infarction and cerebral stroke. Use of drugs is defined as dispensed prescription during the last 6 months

## Discussion

In this large population-based study, high levels of anxiety and depression were associated with UI, strongest for mixed and severe UI. The prevalence of UI was higher among users of several psychotropics compared to non-users. After adjustments, antidepressants were still associated with UI. However, the associations between anxiety/depression and UI were not influenced by use of psychotropic drugs in the present study.

The strengths of the study include a large sample size, a good response rate and a population-based design. All women ≥20 years in the county of Nord-Trøndelag in Norway were invited, in contrast to many other studies on UI only focusing on elderly or other limited age-groups. The study population of Nord-Trøndelag is regarded to be representative of the female population of Norway, and we can assume that it gives representative knowledge about a western, female population. The questions about UI, anxiety and depression were part of a questionnaire covering many medical topics, and this may reduce the risk of underreporting because of embarrassment and overreporting because of eagerness to tell. The scales for both UI, depression and anxiety are well validated. The UI questions are based on the definitions from International Continence Society [19], and includes information making it possible to categorize for both UI type and UI severity. The severity index is well validated [20, 21]. The HADS is widely used in population-based surveys, and the cut-off of 8 has been found to match well with clinically diagnosed anxiety and depression according to DSM-III/IV and ICD-8/9 diagnostic criteria [24]. NorPD contains all dispensed prescriptions for all the women included in the study, and thus reduces the number of missing individuals in the analyses. It has been hypothesized in some studies that urgency is part of a central sensitization that also is an explanation-model for chronic pain [30]. In this study we adjusted for chronic pain and for medication used for it.

Limitations of the study include that questionnaires were returned by mail, with potentially lower participation rate of persons with largest symptom load, e.g. depressed persons. Many of the invited persons in HUNT3 did not come to the screening station or did not return Q1, representing a possible selection bias. Even if HADS is validated as a good instrument for assessing symptom load, it is not a diagnostic tool. A possible bias is also that NorPD only gives information about dispensed prescriptions, not actual use. We had no available information about socioeconomic status, such as income and work. These factors are related to anxiety and depression, but in most studies not to UI, and they are therefore not regarded as confounders. However, a possible impact on our results cannot be excluded, and this may represent a limitation of the study.

The associations between anxiety and UI and depression and UI, especially urgency and mixed type, correspond well with earlier cross-sectional studies [1, 31]. In a population-based survey of 3536 women, major depression was associated with severe UI [2]. Other cross-sectional studies have also shown associations, but the prevalence of UI, depression, and anxiety vary due to different definitions of UI and different cut-offs and definitions of anxiety and depression [32–35]. In a recent study, UI during and after pregnancy was associated with postpartum depression, OR 3.81 (1.57–9.25) [36]. In our previously conducted cross-sectional study of middle-aged women, we found ORs for anxiety and depression of 1.59 (1.36–1.86) and 1.64 (1.32–2.04), respectively, among women with any UI compared to continent women [3]. For severe UI, the ORs for anxiety and depression were 2.30 and 2.14, respectively. The results in the present study thus correspond well with this, but we now also demonstrate an increasing strength of the association by increasing severity of the HADS-A- and HADS-D-score. As far as we know, this is the first study investigating the association for different severity grades of both UI, anxiety, and depression.

Longitudinal studies have shown an association between anxiety and depression at baseline and incident UI. UI at baseline is either not, or weaker, associated with incident anxiety and depression [6, 7]. One longitudinal, population-based study found that UI with condition-specific functional loss predicted incident anxiety disorder [37]. In one study, incontinent women with depression reported a greater functional loss related to their UI than women with UI without depression. Patients with UI and depression also rated their UI as more severe and had greater quality of life impairment [5]. However, the longitudinal studies give stronger support for depression and anxiety leading to UI than UI leading to depression and anxiety.

There are both psychological and biological explanation models for the associations between anxiety/depression and UI. Living with a condition associated with shame, loss of control, unpredictability and decreased quality of life, may lead to psychological stress, anxiety and depression symptoms [35].

Biological theories for the association between anxiety/depression and UI are linked to the serotonergic and noradrenergic systems. Dysregulation of 5-HT and NA in the brain is strongly associated with depression and anxiety. Serotonergic activity inhibits voiding, by inhibiting the parasympathetic input to the bladder and enhancing the efferent control of the urethral outlet. Low levels of 5-HT and NA in the CNS could therefore lead to UI [12]. Peripherally, stimulation of the 5-HT4 receptors in the bladder detrusor, may cause detrusor overactivity and potentially urgency UI. This could be one reason for the association between use of antidepressants and higher prevalences of UI [14, 38].

Different psychotropics interfere in these pathways and earlier studies have investigated a possible association with UI. One study found a relative risk (RR) of 1.9 for UI during use of SSRI [14]. Another study found much higher prevalence of UI among antidepressant users (64%) than in the control group (33%) [26]. There has been some evidence for an association between benzodiazepines and UI [28] and between antiepileptics and UI [27]. Parkinson disease can cause bladder dysfunction and incontinence [39], but there is lack of studies on the effect of antiparkinson-drugs on UI.

The association between the use of psychotropics and UI has previously been investigated in the same data material from HUNT3, and the selection of drugs in our model were chosen partly based on the results in this study [16]. Mauseth et al. found adjusted associations between use of SSRIs and UI and between use of lamotrigine and UI, with ORs 1.52 and 2.73, respectively, for two or more prescriptions during the last 6 months. They also found an association between one dispensed antipsychotic during the last 6 months and UI with OR 1.91, not significant for two or more dispensed prescriptions. They did not find any associations between benzodiazepines or zopiclone/zolpidem and UI. The results for lamotrigin are uncertain, however, because of very few women using this drug. For SSRIs, adjustments for high score on HADS-D in the association did not change the OR considerably. This could indicate that there is an independent association between the drug and UI. Like Mauseth et al., we also found an independent association between antidepressants and UI. However, the association was not present when the women with depression or anxiety using antidepressants were compared with women with these conditions not using antidepressants. Use of anxiolytic medication among patients with anxiety seems to give a small decrease in prevalence of UI. One other study found an association between atypical antipsychotics and lower urinary tract symptoms (LUTS) [27]. Our study did not support such association.

## Conclusions

Anxiety, depression, and UI are common conditions and share hormonal and neurological pathways, where also psychotropic drugs, especially antidepressants, act. There is therefore a biological substrate behind the observed associations in this field. Our study expands the cross-sectional evidence that anxiety and depression are associated with all types of UI in women, with increasing strength of the association by increasing severity of the conditions. Antidepressant drugs seem to have an independent association with UI, but the associations between UI and anxiety/depression were not influenced by use of any psychotropic drug.

## Data Availability

The data used in this study are available from The HUNT databank and The Norwegian Prescription Database, but restrictions apply to the availability of these data. The data were used under license for the current study, and so are not publicly available. However, data are available from the authors upon reasonable request and with included permission from HUNT, The Norwegian prescription database, the Regional Ethical Committee, and Norwegian Data Protection Authority.

## Abbreviations

ATC: Anatomical Therapeutic Chemical
DDD: Defined daily dose
EPINCONT: Epidemiology of incontinence in Nord-Trøndelag
HADS-A: The anxiety part of the Hospital anxiety and depression scale
HADS-D: The depression part of the Hospital anxiety and depression scale
LUTS: Lower urinary tract symptoms
OR: Odds ratio
Q1 and Q2: Questionnaire 1 and questionnaire 2
RR: Relative risk
SSRI: Selective serotonin reuptake inhibitor
UI: Urinary incontinence
